# Toxin-mediated gene regulatory mechanism in *Staphylococcus aureus*

**DOI:** 10.15698/mic2017.01.553

**Published:** 2016-12-29

**Authors:** Hwang-Soo Joo, Michael Otto

**Affiliations:** 1 Laboratory of Bacteriology, National Institute of Allergy and Infectious Diseases, National Institutes of Health, Bethesda, MD 20814, USA.

**Keywords:** Staphylococcus aureus, phenol-soluble modulins, toxin, gene regulation, GntR, YtrA

## Abstract

The dangerous human pathogen *Staphylococcus aureus* relies heavily on toxins to cause disease, but toxin production can put a strong burden on the bacteria’s energy balance. Thus, controlling the synthesis of proteins solely needed in times of toxin production represents a way for the bacteria to avoid wasting energy. One hypothetical manner to accomplish this sort of regulation is by gene regulatory functions of the toxins themselves. There have been several reports about gene regulation by toxins in *S. aureus*, but these were never verified on the molecular level. In our study published in MBio [Joo *et al.*, 7(5). pii: e01579-16], we show that phenol-soluble modulins (PSMs), important peptide toxins of *S. aureus*, release a repressor from the promoter of the operon encoding the toxin export system, thereby enabling toxin secretion. This study describes the first molecular regulatory mechanism exerted by an *S. aureus* toxin, setting a paradigmatic example of how *S. aureus* toxins may influence cell functions to adjust them to times of toxin production.

*S. aureus* is a dangerous human pathogen, whose virulence potential relies on a large series of toxins that interfere with virtually any aspect of survival in the human host. Most notably, it produces efficient toxins to lyse leukocytes, as leukocytes represent the most important arm of innate host defense to eliminate invading staphylococci. Among those are the classical, receptor-dependent leukotoxins, such as the Panton-Valentine leukocidin, and the phenol-soluble modulins (PSMs), which lyse cell membranes, including those of leukocytes, in a receptor-independent fashion based on their membrane-destructive, detergent-like properties.

Gene regulation by *S. aureus* toxins has been reported previously for the *S. aureus* toxic shock syndrome toxin (TSST), the Panton-Valentine leukocidin (PVL), and alpha-toxin, a key toxin of *S. aureus* that is lytic to many cell types and has multiple other modes of action. However, the underlying molecular mechanisms have never been investigated. What is more, in the case of PVL, a secondary mutation in a global regulator - something that happens frequently in *S. aureus* - could later be made responsible for the observed effects; while in independent research, no gene regulatory effect of PVL was found. Overall, whether *S. aureus* toxins actually have direct gene regulatory functions and how those may work has remained elusive.

The PSMs are distinct from other *S. aureus* toxins not only in their mode of action, but also regarding the fact that they are produced in vast amounts. More than half of the secreted protein mass in *S. aureus* stationary-phase cultures is PSMs. Thus, an efficient PSM export system is required, which we recently identified as a dedicated four-component ABC transporter named Pmt. Remarkably, in the absence of Pmt, PSMs accumulate in the cytosol and lead to cell death.

Thus, times of PSM production represent a fundamental switch in cell physiology. It is important to note in that regard that PSMs are under strict quorum-sensing control and thus produced mainly in early stationary growth phase. Microarray analysis of a mutant, in which all members of the *psm* gene family were deleted, revealed significant changes compared to the isogenic wild-type strain. The most interesting among those were PSM-dependent changes in the PSM secretion system, Pmt. The most likely candidate for a gene responsible for the impact of PSMs on *pmt* expression was that encoded just upstream of the *pmt* genes, which was later named *pmtR* for *pmt* repressor.

In our study, we showed that PSMs bind to PmtR, release it from the promoter DNA, and thereby increase production of Pmt (Fig. 1). Also, in the absence of *pmtR* there was a slight, yet significant impact on cell growth, confirming that the regulatory effect contributes to adjusting energy consumption to varying levels of toxin production. Interestingly, there were specific PSMs that were involved with regulation of *pmt*. One PSM in particular, PSMα4, showed a strong regulatory effect, while in contrast to many other PSMα peptides it lacks cytotoxic function, indicating specification for regulatory versus cytotoxic roles during the evolution of PSMs.

**Figure 1:**
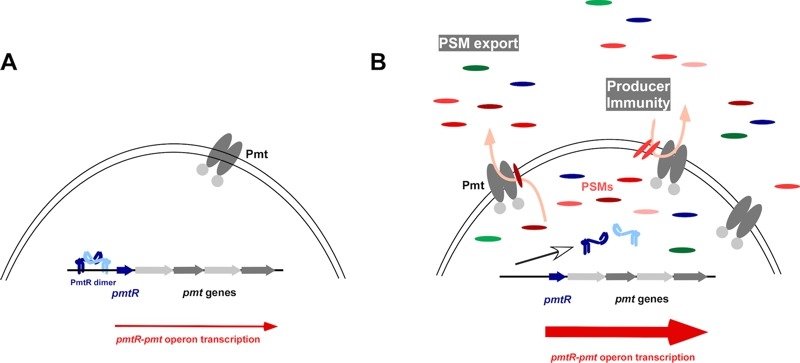
FIGURE 1: PSMs displace PmtR from the *pmt *promoter to ascertain PSM export. **(A)** Without PSMs, the PmtR repressor binds to the *pmt *promoter and strongly decreases transcription of the *pmt* genes coding for the PSM secretion machinery. **(B) **With PSMs, PmtR is displaced from the promoter, allowing for an increase in *pmt *gene transcription. The increased number of Pmt proteins drives PSM export as well as confers protection from the membrane-damaging function of already secreted PSMs (producer immunity).

PmtR belongs to the YtrA subfamily of GntR-type DNA repressor proteins. They all bind DNA as dimers, but what distinguishes YtrA-type proteins is the very small size of the ligand binding domain, which some have pointed out may be too small to actually bind a ligand. In that regard it is interesting that our findings suggest that PSMs interact directly with DNA in a non-specific manner, possibly indicating another mechanism of ligand-PmtR interaction that is distinct from the one that is based on binding to the canonical, distal binding domain. Rather, PSMs may disrupt DNA-PmtR interaction by intercalating between the DNA-binding region of PmtR and the DNA (with the specificity of interaction being determined by the PmtR binding site) (Fig. 2). Which of these two possible mechanisms PSMs use - (i) binding distally, leading to a conformational change that releases the PmtR dimer from the DNA, or (ii) intercalating between PmtR and DNA, will need to be addressed in the future. Future research directions also include analyzing additional regulatory targets of PSMs. While microarray analysis suggested that PmtR is very specific, inasmuch as it only binds to the *pmt* promoter, PSMs appear to also regulate other target genes. Whether this works by a similar mechanism and additional repressor proteins that interact with PSMs, or in a completely different fashion, remains to be determined.

**Figure 2:**
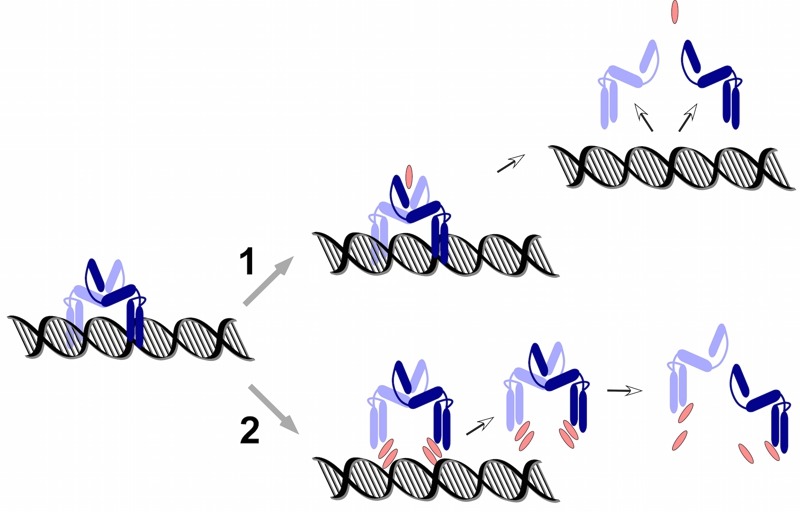
FIGURE 2: Possible mechanisms of PmtR-PSM interaction. **(1)** depicts the canonical mechanism of PSM (red) interaction with the ligand binding pocket, which is located in the distal parts of the interacting PmtR monomers (blue) in the PmtR dimer. **(2) **depicts an alternative mechanism, in which PSMs displace the PmtR dimer by intercalating between the DNA and the DNA-binding domain of PmtR.

Overall, our study demonstrates that direct gene regulatory functions of *S. aureus* toxins exist and provides an example of how they may work on a mechanistic level. Whether other suggested regulatory effects of *S. aureus* toxins, such as most notably those described for TSST and alpha-toxin, also depend on direct interaction with DNA-binding proteins remains to be investigated.

